# Higher IL-6 and IL-4 plasma levels in depressed elderly women are influenced by diabetes mellitus

**DOI:** 10.47626/2237-6089-2022-0466

**Published:** 2024-02-06

**Authors:** Natália S. Dias, Antônio L. Teixeira, Breno S. Diniz, Erica L. Vieira, Bernardo de M. Viana, Izabela G. Barbosa

**Affiliations:** 1 Programa de Pós-Graduação em Neurociências Instituto de Ciências Biológicas Universidade Federal de Minas Gerais Belo Horizonte MG Brazil Programa de Pós-Graduação em Neurociências , Instituto de Ciências Biológicas , Universidade Federal de Minas Gerais (UFMG), Belo Horizonte , MG , Brazil .; 2 Department of Psychiatry and Behavioral Sciences University of Texas Health Science Center at Houston Houston TX USA Department of Psychiatry and Behavioral Sciences , University of Texas Health Science Center at Houston (UTHealth), Houston , TX , USA .; 3 Instituto de Ensino e Pesquisa Santa Casa de Misericórdia de Belo Horizonte Belo Horizonte MG Brazil Instituto de Ensino e Pesquisa , Santa Casa de Misericórdia de Belo Horizonte , Belo Horizonte , MG , Brazil .; 4 Department of Psychiatry, Faculty of Medicine University of Toronto Toronto ON Canada Department of Psychiatry, Faculty of Medicine , University of Toronto , Toronto , ON , Canada .; 5 Geriatric Psychiatry Division Center for Addiction and Mental Health Toronto ON Canada Geriatric Psychiatry Division , Center for Addiction and Mental Health , Toronto , ON , Canada .; 6 Laboratório Interdisciplinar de Investigação Médica UFMG Belo Horizonte MG Brazil Laboratório Interdisciplinar de Investigação Médica , UFMG , Belo Horizonte , MG , Brazil .; 7 Departamento de Saúde Mental Faculdade de Medicina UFMG Belo Horizonte MG Brazil Departamento de Saúde Mental , Faculdade de Medicina , UFMG , Belo Horizonte , MG , Brazil .

**Keywords:** Female, cytokines, inflammation, major depressive disorder, diabetes mellitus, elderly

## Abstract

**Objective:**

This study aimed at investigating a set of peripheral cytokines in elderly female patients with MDD, comparing them to controls, and assessing the potential influence of clinical comorbidities on inflammatory markers.

**Methods:**

Twenty-five elderly female patients diagnosed with MDD and 19 age-matched female controls were enrolled on this study. Plasma levels of interleukin (IL)-4, IL-6, IL-10, interferon (IFN)-γ and tumor necrosis factor (TNF)-α were evaluated with commercially-available assays.

**Results:**

Elderly female patients with MDD exhibited higher plasma IL-6 and IL-4 levels when compared to controls. In a logistic regression model taking cytokine levels, comorbidities, and age into account, only type 2 diabetes mellitus (DM2) remained associated with MDD.

**Conclusion:**

Diabetes influences the association between MDD and higher levels of cytokines in elderly female patients. Future studies should take this evidence into account in order to mitigate confounding factors.

## Introduction

Since the early 1990s, studies have associated depressive disorders with altered levels of circulating inflammatory markers, such as interleukin (IL)-6 and tumor necrosis factor (TNF). ^1 - 3^ This is still an issue under debate, with recent findings confirming the association between altered cytokine levels and MDD. ^4 - 6^ There is also evidence of T helper (Th)1/Th2 cytokine imbalance, ^7 - 10^ with higher Th1/Th2 ratios in depressed patients when compared to controls.

There is emerging literature on inflammatory/immune dysfunction in older adults with depressive disorders. ^11 - 17^ However, most of the literature has failed to control for the role played by medical comorbidities in increasing inflammatory mediators. ^18 , 19^ This is an important issue because depressive disorders are frequently associated with comorbidities that are common in the elderly population and are linked to inflammation, such as type 2 diabetes mellitus (DM2) ^20^ and cardiovascular diseases. ^21^ Depressive disorders are even associated with dementia as a risk factor or as prodrome ^22^ and inflammatory mechanisms probably underly this association. ^23^

The present study aimed to investigate a set of cytokines in elderly female patients with major depressive disorder (MDD), comparing them to controls and exploring potential associations with clinical parameters, including medical comorbidities.

## Methods

### Subjects

This cross-sectional study consecutively included elderly female patients aged 60 years or older at the Universidade Federal de Minas Gerais (UFMG) Psychogeriatrics Outpatient Clinic (Ambulatório de Psicogeriatria) in Belo Horizonte, MG, Brazil. Twenty-five female patients with acute MDD were enrolled. Nineteen age-matched female controls were also enrolled from an ongoing cohort study of healthy cognitive aging at UFMG.

All participants were clinically evaluated with the Mini-International Neuropsychiatric Interview (M.I.N.I. – Plus). ^24^ The severity of depressive symptoms was assessed with the Hamilton Depression Rating Scale 17-item version (HAM-D). ^25^ Participants were excluded if neuropsychological evaluation suggested dementia (scores lower than 2 standard deviation in two or more cognitive domains). The neuropsychological battery comprised the following tests: Mattis Dementia Rating Scale (DRS), ^26^ Digit Span Forward and Backward (DGS), ^27^ Five Digits (FDT), ^28^ Rey Auditory-Verbal Learning Test (RAVLT), ^29^ Verbal Phonemic Fluency, ^30^ Frontal Assessment Battery (FAB), ^31^ Taylor’s Complex Figure Simplified, ^32^ Corsi’s Cubes, ^33^ Clock Drawing Test (CDT), ^34^ Stick Design Test (SDT), ^35^ Laboratory of Neuropsychological Investigations Naming Test, ^36^ and Pfeffer’s Functionality Scale. ^37^ Participants were also excluded if they had mood disorders due to general medical conditions, other psychiatric conditions (except nicotine dependence), infectious or active autoimmune diseases, or if they were using illicit drugs or abusing alcohol. In addition, participants who had used corticosteroids, anti-inflammatories, or antibiotics in the 4 weeks prior to the study were also excluded. Additional exclusion criteria for controls were any psychiatric disorder and use of antidepressant drugs.

Written informed consent was obtained from all participants. The local institutional ethics committee approved the study, which was conducted in accordance with the 1975 Helsinki Declaration.

### Samples

Four milliliters of blood were drawn from each participant by venipuncture into ethylenediamine tetra acetic acid (EDTA) tubes between 8 a.m. and 11 a.m. on the same day of the clinical assessment. Blood was immediately centrifuged at 3,000 rpm and 4 °C for 15 min. Plasma was collected and stored at -80 °C until assayed.

Plasma IL-6, IL-4, IL-10, IFN-γ, and TNF-α levels were measured by Luminex, according to the procedures supplied by the manufacturer of the HCYTOMAG-60k kit (Merck Millipore, Darmstadt, Germany). Plasma cytokine levels for each participant are shown in the supplementary material. Detection limits were defined at 0.9 pg/mL for IL-6, 4.5 pg/mL for IL-4, 1.1 pg/mL for IL-10, 0.8 pg/mL for IFN-γ and 0.7 pg/mL for TNF-α.

### Data analysis and statistical evaluation

Dichotomous variables were assessed with the chi-square test or Fisher’s exact test when the number of cases was ≤ 5 or less than 20% of the group. Non-parametric distribution was considered for continuous variables and they were assessed with the Mann-Whitney U test. The results were presented as medians and interquartile range. IL-4, IL-6, DM2, cardiovascular diseases, hypothyroidism, and age were tested as explanatory/independent variables in the logistic regression model. Variables were included if p was < 0.20 in univariate analyses. The dependent variables were the dichotomous groups “depression” and “control”. Odds ratios (OR) were calculated for statistically significant explanatory/independent variables identified. The omnibus test of model coefficients was used to check if the final model was an improvement over the baseline model. The Cox-Snell R square was used to indicate the model’s percentage of explanation. Statistical tests were two-tailed, considering a significance level of p < 0.05. Statistical analyses were performed using SPSS software version 22.0.

## Results

Demographic and clinical features of all participants are shown in Table 1 . Patients with MDD did not differ from controls in terms of the frequency of cardiovascular diseases or hypothyroidism (p = 0.180 and p = 1.00, respectively). Patients with MDD had a higher frequency of DM2 (p = 0.04).

**Table 1:** Demographics, clinical features, and plasma cytokine levels of elderly female patients with MDD and controls

	Controls (n = 19)	MDD (n = 25)	p-value
Age, median (P25-P75)	65 (62-76)	71 (65-78)	0.122*
HAM-D, median (P25-P75)	1 (0-2)	20 (14-23)	< 0.001*
Medical comorbidities, frequency (%)			
Type 2 diabetes mellitus	2/19 (10.5)	10/25 (40)	0.037 ^†^
Cardiovascular diseases	12/19 (63.2)	19/25 (76)	0.180 ^†^
Hypothyroidism	3/19 (15.8)	5/25 (20)	1.00 ^†^
Cytokines (pg/mL), median (P25-P75)			
IL-6	1.52 (1.12-3.12)	3.04 (1.35-6.94)	0.019*
TNF-α	9.30 (5.34 -12.24)	10.30(7.05-25.55)	0.188*
IL-4	0.34 (0.20-0.65)	0.68 (0.34-1.05)	0.014*
IL-10	1.99 (1.31-4.00)	2.86 (1.48-8.18)	0.180*
IFN-γ	4.27 (2.11-6.29)	6.10 (3.36-13.35)	0.090*
Ratios, medians			
TNF-α/IL-10	3.95	3.58	0.776*
IFN-γ/IL-4	9.40	11.65	0.705*

HAM-D = Hamilton Depression Rating Scale; IFN = interferon; IL = interleukin; MDD = major depressive disorder; P25-P75 = 25%-75% interquartile range; TNF = tumor necrosis factor.

* U Mann-Whitney test.

^†^ Fisher’s exact test.

Median plasma cytokine levels in patients and controls are also shown in Table 1 . Patients had higher plasma IL-6 (p = 0.02) and IL-4 (p = 0.01) levels when compared to controls. There were no significant differences between patients and controls in terms of plasma TNF-α (p = 0.188), IFN-γ (p = 0.090), or IL-10 (p = 0.180). Patients’ TNF-α/IL-10 and IFN-γ/IL-4 ratios did not differ from controls’ (p = 0.78 and p = 0.70, respectively).

Ten patients were taking antidepressant drugs: three of them were taking tricyclic antidepressant drugs and seven of them were taking selective serotonin reuptake inhibitors (SSRI). There were no significant differences between patients taking antidepressants and those not taking these drugs in terms of plasma IL-6 (p = 0.311), IL-4 (p = 0.723), TNF-α (p = 0.892), IFN-γ (p = 0.605), or IL-10 (p = 0.723) levels.

In the logistic regression model of potential predictors of MDD, none of the inflammatory markers (IL-4 [p = 0.62] and IL-6 [p = 0.29]) or any other variables (cardiovascular diseases [p = 0.71], hypothyroidism [p = 0.39] or age [p = 0.05]) remained associated with MDD, with the single exception of DM2 (OR = 6.54; 95% confidence interval [95%CI] = 1.06-57.08; B = 2.05 ± 1.01; p = 0.03). The omnibus test showed that the final model was significantly better fit than the baseline model (p = 0.015), indicating that the accuracy of the model improved when the explanatory variable was added.

## Discussion

In the current study, elderly female patients with MDD exhibited increased levels of immune mediators, but after adjusting for confounding factors, these levels were no longer associated with MDD.

We found higher IL-6 levels in elderly female patients with MDD than the controls. IL-6 has been associated with the pathophysiology of depressive disorders as well as with prognosis and therapeutic response to antidepressants. ^38^ We also found an increase in IL-4 in patients with MDD, which agrees with previous evidence showing a Th2 skewed response in MDD. ^39^ However, in the multivariate analysis, only DM2 remained associated with MDD. There was a significant difference in the frequency of DM2 between patients and controls, which reflects the increased occurrence of DM2 in MDD patients. ^40 - 42^ It is already known that the comorbidity of MDD with DM2 might be related to a broader proinflammatory state, associated both with insulin resistance and with the depressive symptoms. ^43 - 45^ However previous studies failed to control the association of MDD and cytokine levels for DM2 as a confounding factor.

The findings show that cytokine patterns in depressed elderly people may be difficult to clarify due to the pathophysiological processes involved in aging and to the presence of comorbidities. These conditions, particularly DM2, also impact on biomarkers, and thus interfere with any association between depression and inflammatory measures. Nevertheless, our study reinforces the role of cytokines in late-life MDD and comorbidities and calls attention to the need to control for confounders in future studies.

The selection of a sample comprising female patients only should be considered a strength of the study, since gender differences have been shown in immunological profiles related to psychiatric disorders. ^46 - 48^

The sample size constitutes a limitation of the study. This was a convenience sample. Considering this specific age group with strict inclusion and exclusion criteria, the number of participants resulted in the total sample size presented. This sample size was not very different from another recent group and subgroup analysis with a psychogeriatric population. ^49 , 50^ It is also important to consider that sample size must have a relationship with future predictable performance that is fit for purpose and this varies from application to application. ^51^ Despite this limitation, the influence of comorbidities on inflammatory markers of late-life MDD is an important and frequently neglected topic and needs to be investigated.

There are other limitations worth mentioning. Half of the patients were already medicated prior to enrollment in the study. Although there was no difference in cytokine levels between medicated and non-medicated patients, it is not possible to completely rule out the effect of antidepressants. There is also the uncertain relationship between peripheral and central nervous system (CNS) cytokine levels. Correlations between peripheral cytokine levels and cytokine levels in the CNS are still a matter of investigation and debate. ^52 - 54^ It was not possible to distinguish between patients with early-onset and late-onset depression in the present study. Notwithstanding, there is little evidence of distinct cytokine profiles in early-onset and late-onset depression. ^55^ Nutritional status was not controlled in the present study and may interfere in the participants’ inflammatory profile. ^56 , 57^

In conclusion, our findings suggest that higher plasma IL-6 and IL-4 levels in depressed elderly women than in controls are influenced by DM2. More studies are required in order to investigate the present findings. Studies about depression and inflammation should take this evidence into account in order to mitigate confounding factors.
