# Supplementary material for: Higher IL-6 and IL-4 plasma levels in depressed elderly women are influenced by diabetes mellitus
Source: Trends Psychiatry Psychother. 2024 Feb 6;46:e20220466. doi: 10.47626/2237-6089-2022-0466 (PMC11140769; doi:10.47626/2237-6089-2022-0466)
Supplement: Supplementary file 1 [file 2238-0019-trends-46-e20220466-suppl01.pdf]

http://doi.org/10.47626/2237-6089-2022-0466


## Supplementary Material

### Plasma cytokine levels for each participant (pg/mL)

| | IL-6 | IL-4 | Il-10 | IFN-γ | TNF-α |
|---|---|---|---|---|---|
| Patient 1 | 1.32 | 0.59 | 1.48 | 3.62 | 10.01 |
| Patient 2 | 20.27 | 48.51 | 31.01 | 146.42 | 47.47 |
| Patient 3 | 2.00 | 0.34 | 2.7 | 2.56 | 13.3 |
| Patient 4 | 2.28 | 3.62 | 3.29 | 5.79 | 11.08 |
| Patient 5 | 1.25 | 0.22 | 1.66 | 9.17 | 5.95 |
| Patient 6 | 5.79 | 0.31 | 1.57 | 7.05 | 8.77 |
| Patient 7 | 1.56 | 0.59 | 1.48 | 3.11 | 8.39 |
| Patient 8 | 3.04 | 5.2 | 4.48 | 5.48 | 6.75 |
| Patient 9 | 3.52 | 0.72 | 8.49 | 22.41 | 20.78 |
| Patient 10 | 1.35 | 0.68 | 1.48 | 5.2 | 5.48 |
| Patient 11 | 6.22 | 0.88 | 7.87 | 73.2 | 21.86 |
| Patient 12 | 5.94 | 0.37 | 10.00 | 13.04 | 30.63 |
| Patient 13 | 3.00 | 0.68 | 5.4 | 13.04 | 23.28 |
| Patient 14 | 34.92 | 34.11 | 25.43 | 248.93 | 37.38 |
| Patient 15 | 21.1 | 0.78 | 1.44 | 56.26 | 15.01 |
| Patient 16 | 1.22 | 0.26 | 1.99 | 2.35 | 7.36 |
| Patient 17 | 3.3 | 1.01 | 14.2 | 6.1 | 39.92 |
| Patient 18 | 7.67 | 1.1 | 12.06 | 7.73 | 31.01 |
| Patient 19 | 1.12 | 0.31 | 1.31 | 1.5 | 4.94 |
| Patient 20 | 12.94 | 2.08 | 4.59 | 12.24 | 10.3 |
| Patient 21 | 1.63 | 0.29 | 1.18 | 1.73 | 5.95 |
| Patient 22 | 9.72 | 0.34 | 1.33 | 5.34 | 7.69 |
| Patient 23 | 3.08 | 0.55 | 2.87 | 4.27 | 7.47 |
| Patient 24 | 1.35 | 0.72 | 2.87 | 13.67 | 4.88 |
| Patient 25 | 1.25 | 0.37 | 1.66 | 1.95 | 27.82 |
| Control 1 | 3.52 | 0.65 | 16.28 | 4.94 | 26.41 |
| Control 2 | 4.36 | 0.47 | 19.77 | 24.18 | 32.54 |
| Control 3 | 0.96 | 0.22 | 0.95 | 2.03 | 3.76 |
| Control 4 | 4.49 | 0.52 | 10.26 | 14.84 | 30.26 |
| Control 5 | 2.28 | 0.83 | 1.99 | 3.92 | 11.9 |
| Control 6 | 2.65 | 0.24 | 2.49 | 5.2 | 10.61 |
| Control 7 | 3.12 | 5.79 | 4.82 | 5.95 | 10.92 |
| Control 8 | 4.18 | 0.47 | 1.31 | 17.25 | 7.26 |
| Control 9 | 1.05 | 0.29 | 1.44 | 1.58 | 9.3 |
| Control 10 | 2.13 | 2.08 | 4.00 | 4.27 | 11.24 |
| Control 11 | 1.12 | 0.08 | 0.83 | 1.65 | 5.34 |
| Control 12 | 1.02 | 0.2 | 1.44 | 2.35 | 8.39 |
| Control 13 | 1.18 | 0.59 | 1.61 | 6.29 | 12.24 |
| Control 14 | 1.12 | 0.68 | 1.18 | 2.68 | 6.12 |
| Control 15 | 1.52 | 0.22 | 1.94 | 6.12 | 4.37 |
| Control 16 | 2.00 | 0.34 | 2.7 | 2.56 | 13.3 |
| Control 17 | 1.45 | 0.18 | 2.14 | 8.15 | 4.48 |
| Control 18 | 1.28 | 0.2 | 2.34 | 2.11 | 6.29 |
| Control 19 | 0.8 | 0.15 | 0.83 | 1.36 | 1.79 |

IFN = interferon; IL = interleukin; TNF = tumor necrosis factor.
